# Causes of recurrence of paediatric inguinal hernia after single-port laparoscopic closure

**DOI:** 10.1007/s00383-023-05573-0

**Published:** 2024-02-02

**Authors:** Chao-Sheng He, Yi Su, Ming-Xue Liu, Yu-Bing Qin, Yan-Hui Ji, Wen-Qian Huang, Gui-Zhen Huang, Guo-Zhu Yang, Zeng-Long Hu, Suo-Lin Li

**Affiliations:** 1 First Affiliated Hospital of Xiamen University, Xiamen, China; 2 Second Hospital of Hebei Medical University, Shijiazhuang, China

**Keywords:** Paediatric inguinal hernia, Laparoscopic hernia repair, Recurrence, Patent processus vaginalis

## Abstract

**Purpose:**

This paper explores the causes of paediatric inguinal hernia (PIH) recurrence after single-port laparoscopic percutaneous extraperitoneal closure (SPLPEC).

**Method:**

From January 2015 to December 2020, the clinical data of 3480 children with PIHs who underwent SPLPEC were retrospectively reviewed, including 644 children who underwent SPLPEC with a homemade single-hook hernia needle from January 2015 to December 2016 and 2836 children who underwent the SPLPEC with a double-hook hernia needle and hydrodissection from January 2017 to December 2020. There were 39 recurrences (including communicating hydrocele) during the 2–5 years of follow-up. The findings of redo-laparoscopy were recorded and correlated with the revised video of the first operation to analyse the causes of recurrence.

**Result:**

Thirty-three males and 6 females experienced recurrence, and 8 patients had a unilateral communicating hydrocele. The median time to recurrence was 7.1 months (0–38). There were 20 cases (3.11%) in the single-hook group and 19 cases (0.67%) in the double-hook group. Based on laparoscopic findings, recurrence most probably resulted from multiple factors, including uneven tension of the ligation (10 cases), missing part of the peritoneum (14 cases), loose ligation (8 cases), broken knot (5 cases), and knot reaction (2 cases). All children who underwent repeat SPLPEC were cured by double ligations or reinforcement with medial umbilical ligament.

**Conclusion:**

The main cause of recurrence is improper ligation. Tension-free and complete PIH ligation are critical to the success of surgery, which requires avoiding the peritoneum skip area and the subcutaneous and muscular tissues. Redo-laparoscopic surgery was suitable for the treatment of recurrent inguinal hernia (RIH). For giant hernias, direct ligation of the internal ring incorporating the medial umbilical ligament (DIRIM) may be needed.

## Introduction

Since the advent of minimally invasive surgery, research and development of laparoscopic instruments have led to laparoscopic procedures being increasingly performed in paediatric surgery. In particular, laparoscopic hernia repair (LHR) transitioned from three-port intracorporeal suture to SPLPEC [1–3]. Although the single-port laparoscopic technique has been optimized for the treatment of PIH, there are still a few children who experience postoperative recurrence, which is difficult for parents and surgeons who have high expectations for the technique to accept. This study collected the clinical data of 39 patients who experienced recurrence after SPLPEC at the First Affiliated Hospital of Xiamen University and explored the possible causes of the recurrence to guide future operations. The findings were reported and discussed as follows.

## Methods

This is a retrospective study in which we reviewed cases of PIH that were repaired by the single-hook and double-hook SPLPEC. Recurrent cases during follow-up were collected and repaired and their outcomes were reviewed. The findings in the videos during repair of recurrences were correlated to the videos of the primary operations and an analysis was done to postulate the possible causes of recurrence after this technique. Between January 2015 and December 2020, 3480 children who received SPLPEC were followed up. This study was approved by the hospital’s Institutional Review Board. The inclusion criteria for primarily repaired cases included unilateral hernia, bilateral hernia, and incarcerated hernia. The exclusion criteria included hydrocele, direct hernia, recurrent hernia, and inguinal hernia with undescended testis. The patients’ ages ranged from 1 month to 14 years, with 2374 males and 1106 females. Among them, 644 patients underwent the procedure with a homemade single-hook hernia needle from January 2015 to December 2016, and 2836 patients underwent the procedure with a double-hook hernia needle and hydrodissection from January 2017 to December 2020. There were 2229 unilateral and 1251 bilateral cases (including contralateral patent processus vaginalis). Follow-up ranged from 24 to 60 months (32.5 ± 3.2 months), symptoms of recurrence included intermittent inguinal or scrotal swelling (including communicating hydrocele), and 39 cases were confirmed by ultrasonography. All the recurrences were unilateral, of which 8 cases were communicating hydrocele (1 case was formed after ligation of the contralateral patent processus vaginalis). The 39 cases were cured by redo-laparoscopic surgery, and the causes of recurrence were analysed. The primary outcome of our study is to postulate the possible causes of recurrence after PIH repair using the SPLPEC technique, while the secondary outcomes are incidence of recurrence after SPELPEC technique, age and sex distribution of recurrences, and the outcome of management of recurrent cases by double ligation and DIRIM.

### SPLPEC by single-hook

Single-hook SPLPEC was performed as described previously by Suolin Li et al. [4]. A homemade single-hook hernia needle is similar to an epidural puncture needle. A modified 3 mm 30° laparoscope was introduced through an umbilical trocar. The single-hooked needle is passed through the subcutaneous tissues and muscles of the abdominal wall, entering into the extraperitoneal space. The needle further is advanced along the medial side of the internal inguinal ring (IIR) , separating the vas deferens from the peritoneum, penetrating the posterior peritoneum into the abdominal cavity, then the core of the needle is withdrawn. A 2–0 nonabsorbable suture was introduced into the needle sheath, and was sent to 5–8 cm into the abdominal cavity. The hernia needle was then withdrawn, punctured through the abdominal wall once again, passed around the lateral half of the IIR, separating and crossing the testicular vessels, and pushed through the previous peritoneal puncture point to enter the abdominal cavity again. The needle core was pushed out to hook the reserved suture tail and pull it out of the body. Then, both suture ends were stretched to ligate the IIR, and the knot was embedded under the skin. The same steps would be needed if there was contralateral patent processus vaginalis.

### SPLPEC by double-hook

SPLPEC by double-hook was performed as described previously by H Yonggang et al. [5]. The back end of the double-hook hernia needle was connected to a syringe to inject 3–5 ml of saline to separate the peritoneum fold from the vas deferens and passed medially to puncture the peritoneum. After leaving a suture in the abdomen, the needle was slowly retreated through the extraperitoneal space without exiting the abdominal wall. Hydrodissection was performed to float the peritoneum away from the testicular vessels on the lateral half. Then, the needle crossed the testicular vessel and approached the same peritoneal puncture point to enter the abdominal cavity again. The tail of suture was grabbed and pulled out through the abdominal wall. The IIR was closed by extraperitoneal ligation. The same steps would be needed if there was contralateral patent processus vaginalis.

### RIH reoperation

The laparoscopic procedure was the same as SPLPEC described above. We compared the original video with findings at second laparoscopic look for all 39 cases. We speculated on the possible causes of recurrence through the structural abnormalities of the IIR. The status of both internal rings, location of the knot, cause of RIH, size of the IIR, and the presence of an omental adhesion were carefully examined. The IIR was closed by double ligation with a 2–0 nonabsorbable suture. In particular, the second ligation included the first ligation to ensure complete closure of the IIR. A giant hernia may need DIRIM [6].

## Results

Thirty-nine recurrences were unilateral. Thirty-one cases (hernias) that were confirmed via ultrasonography had omentum or intestine involvement, 8 cases (communicating hydroceles) had liquid dark areas, and 1 patient developed a communicating hydrocele after loose ligation of the patent processus vaginalis. Twenty-one cases were on the left and 18 cases were on the right. The median time to recurrence was 7.1 months (0—38). There were 33 males and 6 females, with 20 cases in the single-hook group and 19 cases in the double-hook group (Table 1). Thirty-one patients were younger than 5 years old, 7 patients were between 6 and 10 years old, and only 1 patient was older than 10 years old. These differences in age distribution among recurrences are significant (*P* = 0.031) (Table 2). There was a higher recurrence rate for children 0–5 years old. All children who underwent repeat SPLPEC were cured by double ligation or DIRIM. The average follow-up period was 29.3 months (21–60 months), and no secondary recurrence was found, which was consistent with the findings of previous scholars [7, 8]. RIH resulted from multiple factors, possible causes of recurrence included uneven tension of the ligation, missing part of the peritoneum, loose ligation, broken knot, and knot reaction (Table 3).

**Table 1:** Characteristics of the children with RIH

Variables	N (%)
Age (months)	40.2 ± 29.7 (0–144)
0–2 years old	15 (38.5%)
2–4 years old	7 (17.9%)
4–6 years old	10 (25.6%)
6–10 years old	6 (15.4%)
> 10 years old	1 (2.6%)
Sex	
Male	33 (84.6%)
Female	6 (15.4%)
Location of the hernia	
Left	21 (53.8%)
Right	18 (46.2%)
Bilateral	0 (0.0)
Needle type	
Single-hook	20 (51.3%)
Double-hook	19 (48.7%)
Median time to recurrence(months)	(7.1 ± 8.9) (0–38)
< 1 year	33 (84.6%)
1–2 years	3 (7.7%)
2–3 years	2 (5.1%)
3–4 years	1 (2.6%)
> 4 years	0 (0.0)

Categorical variables are represented as numbers (%) and continuous variables are represented as the mean ± standard deviation (range)

**Table 2:** Univariate analysis of postoperative recurrence of PIH

Groups	Case (N)	Age(year)			Sex		Needle	
		< 5	6–10	> 10	Male	Female	Single-hook	Double-hook
No-recurrence	3441	2063	1147	231	2341	1100	624	2817
Recurrence	39	31	7	1	33	6	20	19
*X*^*2*^		6.965			4.382		29.750	
*P*		0.031			0.036		< 0.01

**Table 3:** Classification of the causes and number of RIH

View under the laparoscope	Cause of recurrence	Single-hook (N)	Double-hook (N)	Total (N)
No knot was seen outside the IIR, IIR is large	Tension ligation cut peritoneum and slid towards abdominal wall	9	1	10
Knot was seen outside the IIR, IIR is small	Missing or tearing part of peritoneum caused incomplete ligation	6	8	14
Knot was seen inside the IIR, IIR is small	The abdominal wall was thick The ligation was loose	2	6	8
Knot was seen outside the IIR, IIR is large	The ligation line slipped or was broken	1	4	5
Tissue reactions to sutures, subcutaneous abscess in inguinal	Recurrence after cutting out the knot	2	0	2
Total(N)		20	19	39

## Discussion

PIH is the most common surgical condition in paediatric surgery, with an incidence of approximately 0.8 to 4.4% [2]. Simple IIR ligation is a conventional procedure that can be divided into open ligation and LHR, both with good outcomes [2, 3, 9]. However, open surgery in which the hernia sac is separated from the spermatic cord vessels may cause more injuries and may be complicated by scrotal oedema or haematoma. Laparoscopic surgery has replaced conventional open surgery because it is advantageous for exploring the contralateral patent vaginal process, decreasing damage to the vas deferens and testicular vessel, and reducing postoperative pain. LHR can be performed through intracorporeal and extraperitoneal approaches, using three, two, or one cannula, either via in vitro or in vivo ligation [10]. In SPLPEC, the IIR is percutaneously ligated with various instruments that have been improved and popularized because of their ability to cause less trauma, thereby simplifying and improving the efficacy of the procedure and reducing the appearance of scars [2, 4, 5]. However, SPLPEC still has a recurrence rate between 0 and 5.5%, which may vary according to the type of surgery, size of the study population, and follow-up period [11]. By achieving proficiency, mastering the learning curve, and optimizing the operating technique, the recurrence rate decreased to 3.11% in the simple-hook hernia needle group and to 0.67% in the double-hook hernia needle group. The high recurrence rate during our early experience was mainly attributed to the four attending and resident doctors’ lack of experience; however, the recurrence rate was significantly reduced after they mastered the learning curve [3]. The recurrence rate in males is higher than that in females, which can be better understood by considering the differences in the anatomic structure of the IIR. Because the spermatic vessels must be crossed in males, the surgery is more complicated and the recurrence rate is higher. There was a higher recurrence rate for children 0–5 years old who had a thin peritoneal membrane at the IIR that possibly could be easily torn, and their inability to cooperate with postoperative quiet rest increased their abdominal pressures [12, 13]. Combined with the findings of laparoscopic reoperation, the causes of recurrence were analysed to improve the operation specifications, reduce the risk factors for postoperative recurrence [8, 12], and explore the appropriate reoperation methods.

### Tension in the ligation

**Fig. 1:**
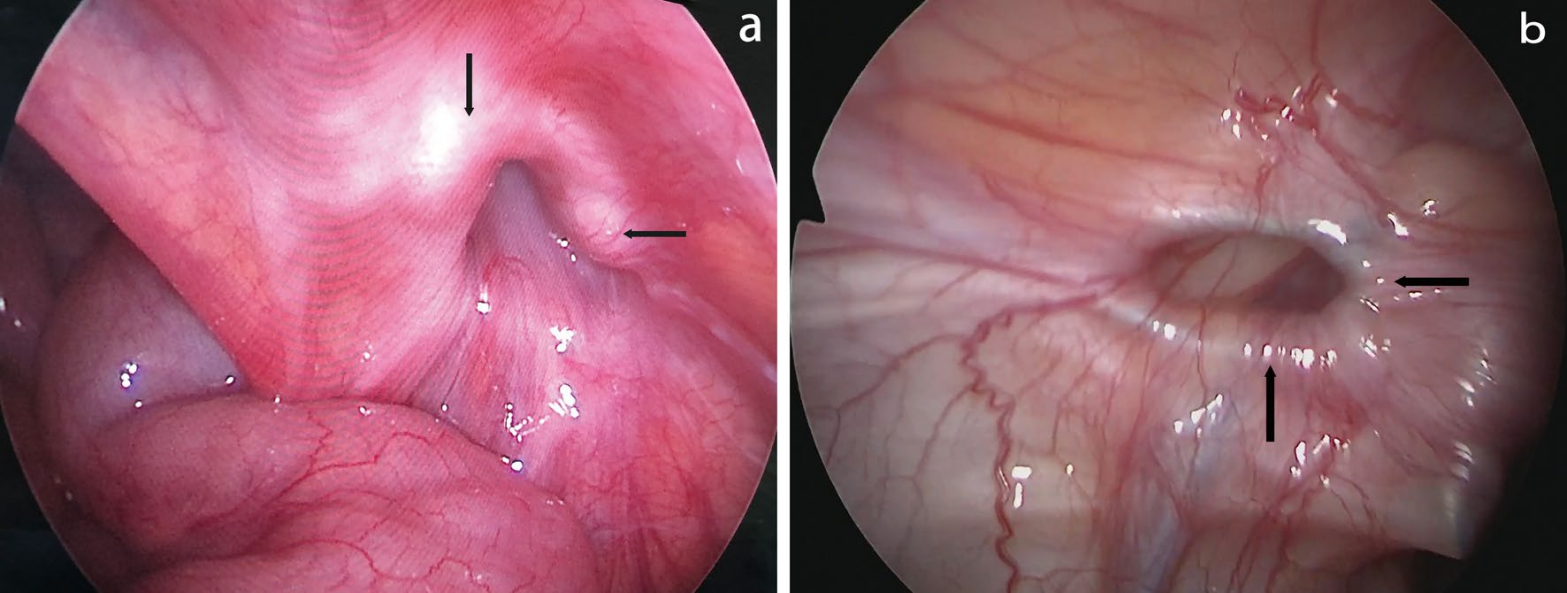
**a**, **b** No obvious knot was seen, and the internal ring showed circumferential thickening (black arrow)

Tension mainly occurred in the single-hook hernia needle group (9 cases). This is likely because the single-hook hernia needle requires two abdominal wall punctures with sending and hooking suture of the IIR. Ligation involving some abdominal wall muscle and fascia tissue is inevitable. The knot can only be tied under the skin. In particular, the thicker abdominal wall of older children provides more tissue for ligation, and cutting the peritoneal membrane as well as movement of the abdominal wall cause recurrence. This is suggested by the presence of a knot outside the IIR with a large ring. The knot sliding to the abdominal wall and the presence of postoperative abdominal wall pain are strong evidence (Fig. 1a, b). Puncturing the abdominal wall with the improved double-hook hernia needle enables the knot to slip in the anterior extraperitoneal space through the muscle layers, allowing tension-free ligation of the IIR and significantly reducing the recurrence rate [2].

### Missing or tearing the peritoneum

**Fig. 2:**
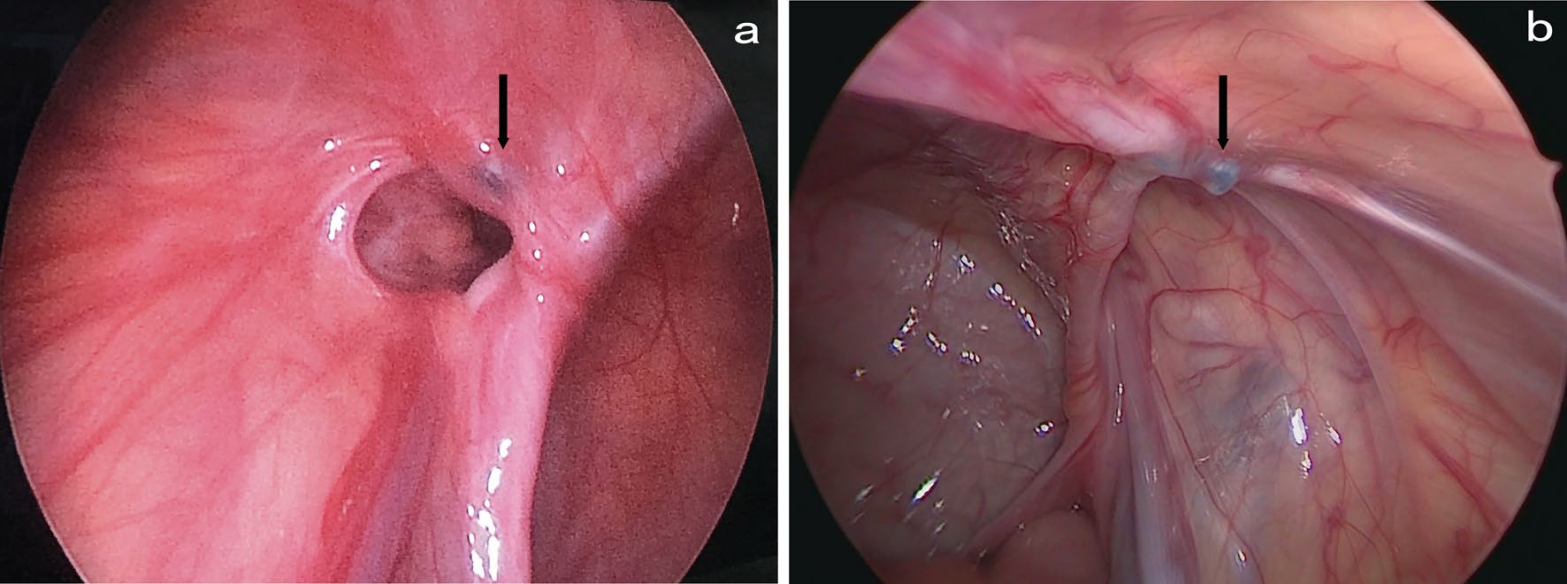
**a**, **b** Knot (black arrow), missing or tearing a part of the peritoneum, inguinal hernia recurred from the posterior side

**Fig. 3:**
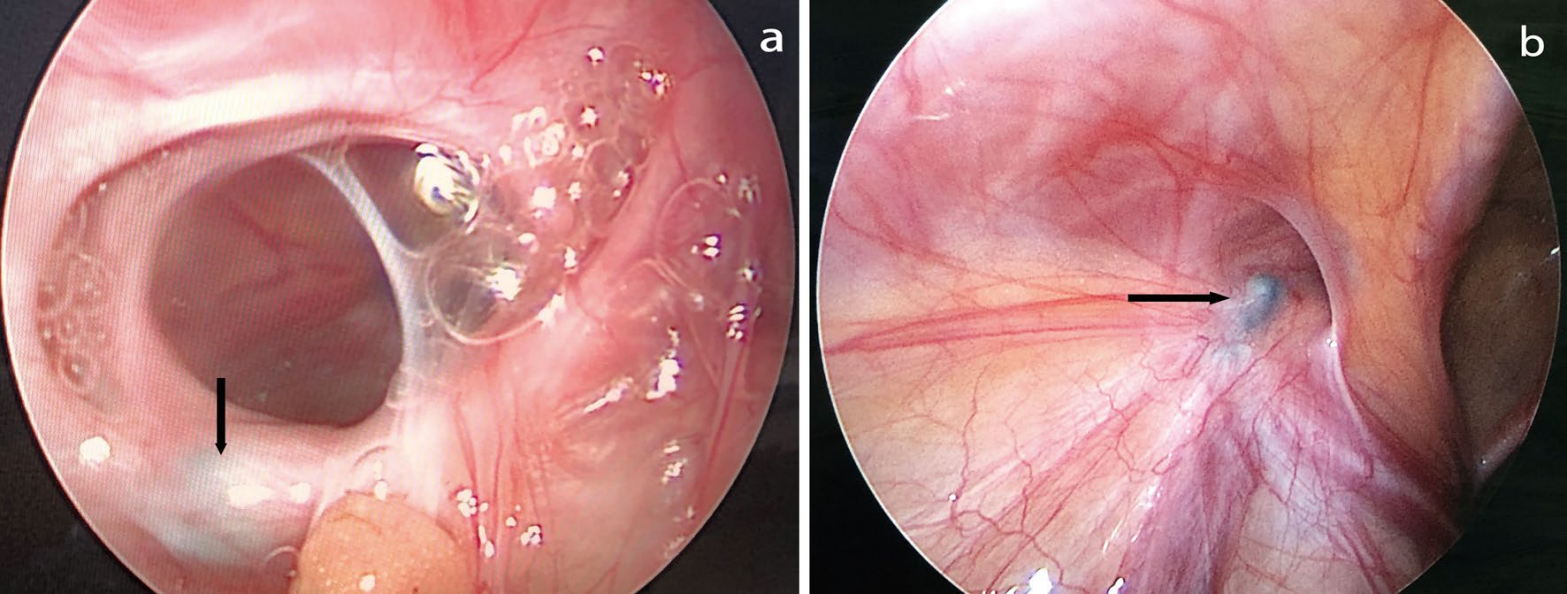
**a**, **b** Knot (black arrow), missing or tearing a part of the peritoneum, inguinal hernia recurred from the anterior side

Six patients in the single-hook needle group and 8 patients in the double-hook needle group had missing or torn parts of the peritoneum. The main reason for consulting the first operation video is to observe the looseness of the IIR with respect to more posterior peritoneal folds. It is difficult to ligate the folded peritoneum with a single needle during laparoscopy. The missing part of the IIR peritoneum could not be ligated completely, thereby changing the “big hole” into the “small hole”. A knot could be seen from the anterior and lateral sides of the IIR in the repeat laparoscopy, with most of the IIR opened on the posterior side of the knot (Fig. 2a, b) and a small portion of the IIR opened on the anterior side of the knot (Fig. 3a, b) [14]. If the peritoneum was missed or torn in the posteromedial side, the recurrent internal ring would be located behind the knot. If the peritoneum was missed or torn in the anteromedial side, the recurrent internal ring would be located in front of the knot. It is easy for beginners who are inexperienced to incompletely tie the IIR. In this case, complete ligation of the IIR peritoneum can be achieved with the assistance of a paraumbilical laparoscopic grasper [4].

### Loose ligation of IIR

**Fig. 4:**
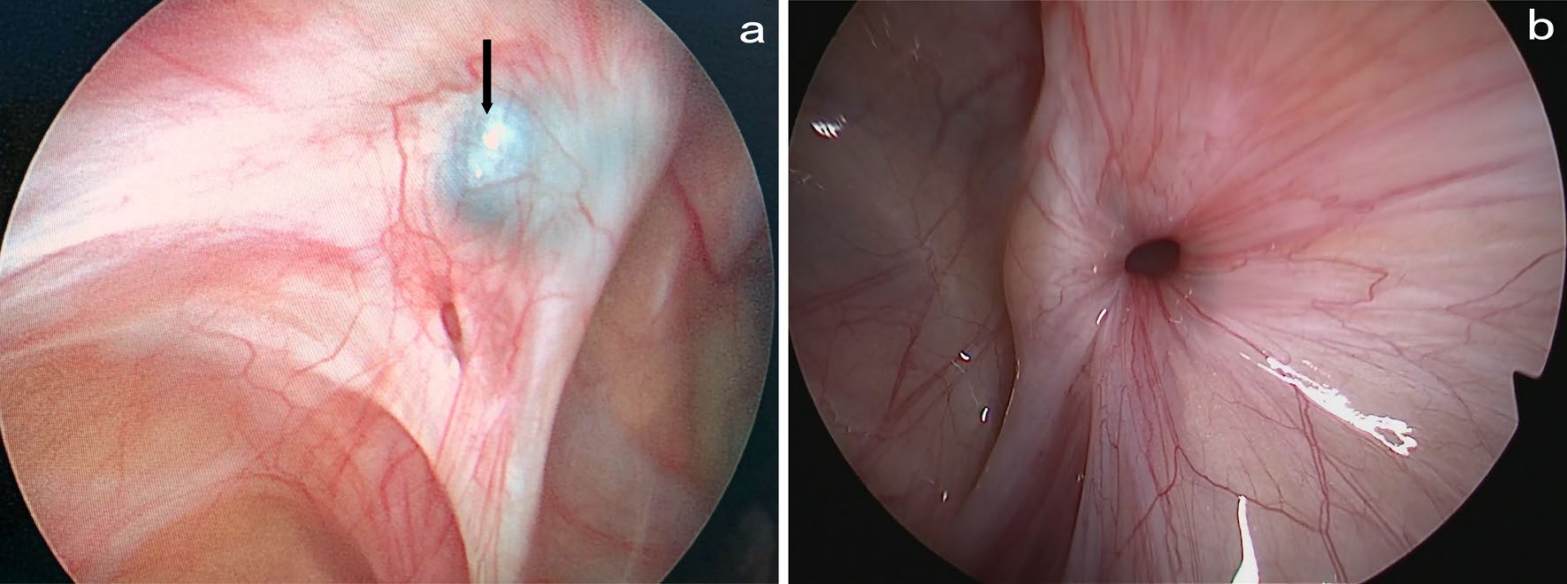
**a**, **b** Knot (black arrow). The internal ring is small

Eight patients had a communicating hydrocele. Under the laparoscope, the IIR was small, approximately 3 mm, with a complete knot. They were all older children with obesity and an abundance of subcutaneous fat. It was difficult to ligate the knot with sufficient tightness in vitro. With a thick abdominal wall, the knot may become a water-drop-shaped knot that slides to the far end, making it difficult to close the IIR (Fig. 4a, b). For this reason, improving the tying skills, using a strong weaving suture (Ethibond), pushing the knot to the extraperitoneal space with a knot pusher, or tying knots by using double ligation (putting the first knot into the second knot) to ensure that the ligation is stable may improve the efficacy of the procedure.

### Slipped or broken ligation line

**Fig. 5:**
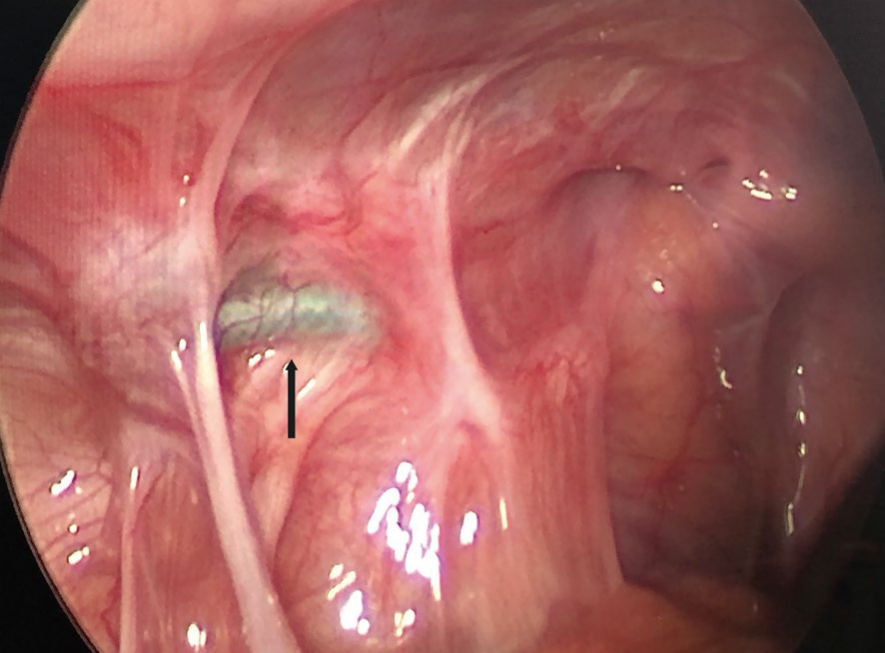
The ligation line is broken (black arrow)

A circuitous suture was observed outside the peritoneum, possibly indicating that untying of the ligation led to recurrence. It is possible that the tying knot was only a square knot, but the suture end was cut too short and became loose, or the suture was broken by the sharp hook (Fig. 5). Therefore, during the operation, one end of the suture should be hung in the needle and pulled out of the abdomen, and the damage-free intermediate line segment should be chosen to tie the IIR. Make sure to tie at least three single or surgical knots, and leave a 2–3 mm suture end to avoid loosening.

### Knot reaction leading to subcutaneous abscess

Two cases occurred in the single-hook hernia needle group, with one case being a communicating hydrocele. Due to a subcutaneous suture knot reaction and **s**ubcutaneous abscess, recurrence occurred after removing the ligation line [15]. Because the surgeon was inexperienced and in the early stages of the learning curve, two punctures of the abdominal wall may not be in the same needle path, and excessive tissue was ligated, thereby leading to ischaemia, necrosis, and inflammation. Therefore, it is necessary to use a double-hook needle to make punctures from both sides of the IIR to pass the same needle path through the peritoneum, to complete extraperitoneal closure, and to avoid a knot reaction. In the follow-up of the double-hook hernia needle group, it was found that the knots located in the extraperitoneal space seldom formed tissue reactions.

## Conclusion

The main cause of recurrence is improper ligation. Tension-free and complete PIH ligation may be critical to the success of surgery, which requires avoiding the peritoneum skip area and the subcutaneous and muscular tissues. Redo-laparoscopic surgery was suitable for the treatment of RIHs. For giant hernias, DIRIM may be needed.

## Data Availability

Data will be made available on request.

## References

[1] Davies DA, Rideout DA, Clarke SA (2020) The international pediatric endosurgery group evidence-based guideline on minimal access approaches to the operative management of inguinal hernia in children. J Laparoendosc Adv Surg Tech A 30:221–227. 10.1089/lap.2016.0453 28140751 10.1089/lap.2016.0453

[2] Li S, Xu W (2018) Endoscopic surgery group, pediatric surgery branch, Chinese medical association. Guidelines for laparoscopic surgery of pediatric inguinal hernia (2017 edition) (above). Chin J Hernia and Abdom Wall Surg (Electronic Version) 12:1–5. 10.3877/cma.j.issn.1674-392X.2018.01.001

[3] Maat S, Dreuning K, Nordkamp S et al (2021) Comparison of intra- and extra-corporeal laparoscopic hernia repair in children: A systematic review and pooled data-analysis. J Pediatr Surg 56:1647–1656. 10.1016/j.jpedsurg.2021.01.049 33674123 10.1016/j.jpedsurg.2021.01.049

[4] Li S, Liu L, Li M (2014) Single-port laparoscopic percutaneous extraperitoneal closure using an innovative apparatus for pediatric inguinal hernia. J Laparoendosc Adv Surg Tech A 24:188–193. 10.1089/lap.2013.0288 24568586 10.1089/lap.2013.0288

[5] Yonggang H, Changfu Q, Ping W et al (2019) Single-port laparoscopic percutaneous extraperitoneal closure of inguinal hernia using “two-hooked” core needle apparatus in children. Hernia 23:1267–1273. 10.1007/s10029-019-01933-9 30993474 10.1007/s10029-019-01933-9

[6] Beytullah Y, Ergun E, Sertaç H et al (2022) Direct ligation of the internal ring incorporating the medial umbilical ligament (DIRIM): a new modification for laparoscopic percutaneous inguinal hernia repair in children. Pediatr Surg Int 38:1083–1088. 10.1007/s00383-022-05131-0 35511252 10.1007/s00383-022-05131-0

[7] Zhu H, Li J, Peng X et al (2019) Laparoscopic percutaneous extraperitoneal closure of the internal ring in pediatric recurrent inguinal hernia. J Laparoendosc Adv Surg Tech A 29:1297–1301. 10.1089/lap.2019.0119 31393202 10.1089/lap.2019.0119

[8] Xiang B, Jin S, Zhong L et al (2015) Reasons for recurrence after the laparoscopic repair of indirect inguinal hernia in children. J Laparoendosc Adv Surg Tech A 25:681–683. 10.1089/lap.2014.0401 26171577 10.1089/lap.2014.0401

[9] Wu S, Xing X, He R et al (2022) Comparison of laparoscope-assisted single-needle laparoscopic percutaneous extraperitoneal closure versus open repair for pediatric inguinal hernia. BMC Surg 22:334. 10.1186/s12893-022-01787-6 36085145 10.1186/s12893-022-01787-6 PMC9461258

[10] Chen Y, Wang F, Zhong H et al (2017) A systematic review and meta-analysis concerning single-site laparoscopic percutaneous extraperitoneal closure for pediatric inguinal hernia and hydrocele. Surg Endosc 31:4888–4901. 10.1007/s00464-017-5491-3 28389795 10.1007/s00464-017-5491-3

[11] Shibuya S, Imaizumi T, Yamada S et al (2022) Comparison of surgical outcomes between laparoscopic percutaneous extracorporeal closure (LPEC) and open repair for pediatric inguinal hernia by propensity score methods and log-rank test analysis. Surg Endosc 36:941–950. 10.1007/s00464-021-08354-9 33616732 10.1007/s00464-021-08354-9

[12] Miyake H, Fukumoto K, Yamoto M et al (2017) (2017) Risk factors for recurrence and contralateral inguinal hernia after laparoscopic percutaneous extraperitoneal closure for pediatric inguinal hernia. J Pediatr Surg 52:317–321. 10.1016/j.jpedsurg.2016.11.029 27894761 10.1016/j.jpedsurg.2016.11.029

[13] Taylor K, Sonderman KA, Wolf LL et al (2018) Hernia recurrence following inguinal hernia repair in children. J Pediatr Surg 53:2214–2218. 10.1016/j.jpedsurg.2018.03.021 29685492 10.1016/j.jpedsurg.2018.03.021

[14] Hayashi K, Ishimaru T, Kawashima H (2019) Reoperation after laparoscopic inguinal hernia repair in children: a retrospective review. J Laparoendosc Adv Surg Tech A 29:1264–1270. 10.1089/lap.2019.0191 31433242 10.1089/lap.2019.0191

[15] Gong D, Qin C, Li B et al (2020) Single-site laparoscopic percutaneous extraperitoneal closure (SLPEC) of hernia sac high ligation using an ordinary taper needle: a novel technique for pediatric inguinal hernia. Hernia 24:1099–1105. 10.1007/s10029-020-02180-z 32266601 10.1007/s10029-020-02180-z

